# Comparative evaluation by semiquantitative reverse transcriptase polymerase chain reaction of MDR1, MRP and GSTp gene expression in breast carcinomas.

**DOI:** 10.1038/bjc.1998.115

**Published:** 1998-03

**Authors:** R. Lacave, F. Coulet, S. Ricci, E. Touboul, A. Flahault, J. G. Rateau, D. Cesari, J. P. Lefranc, J. F. Bernaudin

**Affiliations:** Laboratoire d'Histologie et Biologie Tumorale et UniversitÃ© Pierre et Marie Curie - Paris 6, HÃ´pital Tenon, France.

## Abstract

**Images:**


British Journal of Cancer (1998) 77(5), 694–702
© 1998 Cancer Research Campaign

### Comparative evaluation by semiquantitative reverse transcriptase polymerase chain reaction of *MDR1*, *MRP* and *GSTp* gene expression in breast carcinomas

**R Lacave[1], F Coulet[1], S Ricci[1], E Touboul[2], A Flahault[3], JG Rateau[1], D Cesari[1], JP Lefranc[4] and JF Bernaudin[1]**

[1]Laboratoire d'Histologie et Biologie Tumorale et Université Pierre et Marie Curie – Paris 6, Hôpital Tenon, 4 rue de la Chine, 75020 Paris, France; [2]Oncologie-Radiothérapie, Hôpital Tenon, 4 rue de la Chine, 75020 Paris, France; [3]Biostatistiques et Informatique Médicale, Hôpital Tenon, 4, rue de la Chine, 75020 Paris, France; [4]Chirurgie Gynécologique, Groupe hospitalier Pitié-Salpetrière, 95 Bd de l'hôpital, 75013 Paris, France

**Summary** Identification and quantitative evaluation of drug resistance markers are essential to assess the impact of multidrug resistance (MDR) in clinical oncology. The *MDR1* gene confers pleiotropic drug resistance in tumour cells, but other molecular mechanisms are also involved in drug resistance. In particular, the clinical pattern of expression of the other MDR-related genes is unclear and their interrelationships are still unknown. Here, we report standardization of the procedures used to determine a reliable method of semiquantitative reverse transcriptase polymerase chain reaction (RT-PCR) using a standard series of drug-sensitive and increasingly resistant cell lines to evaluate the expression of three MDR-related genes, i.e. *MDR1* (multidrug resistance gene 1), *MRP* (multidrug resistance related protein) and *GSTp* (glutathione-S-transferase p), reported to be endogenous standard genes for normalization of mRNAs. A total of 74 breast cancer surgical biopsies, obtained before any treatment, were evaluated by this method. When compared with classical clinical and laboratory findings, *GSTp* mRNA level was higher in diploid tumours. However, the main finding of our study suggests a clear relationship between two of these MDR-related gene expressions, namely *GSTp* and *MRP*. This finding provides new insight into human breast tumours, which may possibly be linked to the glutathione conjugate carrier function of MRP. Well defined semiquantitative RT-PCR procedures can therefore constitute a powerful tool to investigate MDR phenotype at mRNA levels of different related genes in small and precious tumour biopsy specimens.

**Keywords:** chemoresistance; breast carcinoma; *MRP*; *MDR1*; *GSTp*; reverse transcriptase polymerase chain reaction; glutathione S conjugate carrier

A wide variety of cell changes, either spontaneous or induced by cytotoxic exposure, can lead to multidrug resistance phenotype (MDR) (Simon and Schindler,1994). They are frequently interrelated and may coexist in a population of tumour cells. Some proteins have been demonstrated to be overexpressed in MDR cell lines, defining a group of MDR-related genes. The first of these proteins to be identified was P-glycoprotein (P-gp), product of the *MDR1* (multidrug resistance gene 1) (Endicott and Ling, 1989; Gottesman and Pastan, 1993) gene in man, belonging to the ATP-binding cassette (ABC) proteins superfamily, all members of which are membrane transporters. The multidrug resistance related protein (MRP), which has been recently demonstrated to be involved in the MDR phenotype (Cole et al, 1992; Slovak et al, 1993), is a 190 kDa protein, which also belongs to the ABC transporter superfamily. Like P-gp, MRP, which is mainly located in the plasma membrane of resistant cells (Flens et al, 1994; Zaman et al, 1994; Almquist et al, 1995) acts by extruding drugs from the cells (Breuninger et al, 1995). Among other proteins frequently overexpressed in tumour cells presenting a MDR phenotype, GSTp (glutathione-S-transferase p) has been extensively implicated (Moscow et al, 1989; Tew, 1994), although its precise role in the MDR phenomenon has not yet been fully clarified.

*Received 30 April 1997*
*Revised 11 August 1997*
*Accepted 12 August 1997*

*Correspondence to:* R Lacave, Laboratoire d'Histologie et Biologie Tumorale, Hopital Tenon, 4 rue de la Chine, 75970 Paris Cedex 20, France

The development of drug resistance marker evaluation in clinical samples is therefore particularly worthwhile to lead subsequently to prospective clinical correlative studies. Transcriptional rate measurement of the genes involved in MDR constitutes a first step to delineate the in vivo mechanisms used by tumour cells to acquire clinical MDR characteristics. To date, partly due to high material consuming methods, the majority of investigations in patients have focused on a single parameter, mainly P-gp expression. Multiparameter studies, which are not always easily performed on small tissue samples, can use low material consuming methods such as reverse transcriptase polymerase chain reaction (RT-PCR) or RNAase protection assay to simultaneously screen the transcriptional rate of sets of genes involved in MDR.

The main goal of the present study was, therefore, to develop a technically valid method able to investigate the coexpression of three MDR-related genes, i.e. *MDR1*, *MRP*, and *GSTp* on small tumour specimens. The first step in this process, using RT-PCR, was to establish standard curves to semiquantitatively measure the expression of drug resistance genes in control cell lines. They were independently ascertained and their respective precise experimental procedures are reported here.

In the second part of this study, we used this method to screen the MDR phenotype of a consecutive series of 74 untreated invasive breast carcinomas and to compare the levels of expression of each of the three genes with the other two drug resistance genes. The MDR phenotype appeared independent of other clinical, histopathological and laboratory parameters, except that diploid tumours exhibited a higher level of *GSTp* expression than aneuploid tumours. However, our study provides a new intriguing clue in the clinical investigation of MDR, by demonstrating, for the first time in human tumour samples, a clear relationship between *MRP* and *GSTp* gene expression. This finding needs to be discussed in the light of the considerable recent experimental evidence (Jedlitschky et al, 1994; Müller et al, 1994; Zaman et al, 1995) concerning the role of the membrane associated MRP pump in the export of glutathione S conjugates (GS-X) from cells.

### MATERIALS AND METHODS

### Patients and tissue samples

Seventy-four tumour samples were obtained during surgery from previously untreated primary breast cancers. All patients gave their informed consent before surgery. Samples (100 mg to 1 g) were removed from the tumour zone, immediately snap frozen and stored in liquid nitrogen, then cut into several equal pieces for the various analyses. Representative samples were examined histologically to ensure very large predominance of tumour over stromal cells (above 90%) in samples used for analysis. Histopathological typing, Scarff–Bloom–Richardson (SBR) grading and measurements of oestrogen (ER) and progesterone (PR) receptor levels (cut-off values 10 fmol $mg^{-1}$ protein) were performed by other independent investigators (Pathology Department – hôpital Pitié-Salpétrière, Professor Lecharpentier and Biochemistry Laboratory – hôpital Bicêtre, Professor Milgrom) in a tumour area very close to the surgical specimen.

### Flow cytometry procedures

Flow cytometry was performed after DNA labelling with propidium iodide (Sigma Chemical, St Louis, MO, USA). A single-cell suspension was prepared from each tumour sample by mechanical disaggregation, as described previously (Coste et al, 1996). Cellular DNA content was analysed on an Epics Elite flow cytometer (Coulter Electronics, Hialeah, FL, USA). Debris and clumps were eliminated and the results were recorded on 15 000 cells.

**Table 1** Designation of RT-PCR primers used for amplification of both MDR-related genes and internal standards

| Transcript | Primer sequence (5′-3′) | Fragment length |
|---|---|---|
| MDR1 | CCCATCATTGCAATAGCAGG<br>GTTCAAACTTCTGCTCCTGA | 167 |
| β2m | ACCCCCACTGAAAAAGATGA<br>ATCTTCAAACCTCCATGATG | 120 |
| MRP | GGACCTGGACTTCGTTCTCA<br>CGTCCAGACTTCTTCATCCG | 291 |
| GSTπ | CTCCGCTGCAAATACATCTC<br>ACAATGAAGGTCTTGCCTCC | 137 |
| PGK | CAGTTTGGAGCTCCTGGAAG<br>TGCAAATCCAGGGTGCAGTG | 247 |

### Control tumour cell lines

The drug-sensitive human epidermoid carcinoma KB 3.1 cell line and its multidrug-resistant derivative KB 8.5 cell line were kindly donated by M Gottesman (Akiyama et al, 1985). MCF7 human breast carcinoma cells (Soule et al, 1973) were obtained from J Robert, who selected a doxorubicin-resistant cell line (MCF7R) from MCF7S wild-type cells. The IGROV cell line was a gift from J Bénard (1985).

Cell culture media and their supplements were obtained from Gibco BRL (Eragny, France). The KB cell lines were maintained in Dulbecco's modified Eagle medium, while MCF7 and IGROV cell lines were grown in RPMI 1640 medium. All were supplemented with 1.5 mM glutamine, 10% fetal calf serum, 50 IU $ml^{-1}$ penicillin and 50 mg $ml^{-1}$ streptomycin. Cells were cultured at 37°C in a humidified atmosphere containing 5% carbon dioxide. The selection pressure was maintained on resistant cell lines by the addition of either 10 ng $ml^{-1}$ of colchicine or 2 mM doxorubicin for KB 8.5 and MCF7R respectively.

### Semiquantitative RT-PCR analysis

Semiquantitative determination of *MDR1*, *GSTp* and *MRP* gene expression was assessed on a single tissue sample per patient. As proposed by Noonan et al (1990) and as more recently recommended (Beck et al, 1996), we used the β2-microglobulin (β2) gene as the internal control sequence for *MDR1* analysis. As the MCF7R cell line did not express β2, *MRP* and *GSTp* gene expression was monitored using the phosphoglycerate kinase (*PGK*) gene as endogenous standard (Ozcelik et al, 1995).

Total RNA was extracted from either control cell lines or tissue samples using RNA BTM protocol (Bioprobe Systems, Montreuil, France), according to the manufacturer's instructions. RNAs were quantitated by absorbance spectrophotometry measurements and their quality was estimated after migration on a 2% agarose gel.

A 2-µg sample of total cellular RNA from each adequate RNA preparation was treated in a 10-µl volume, with 2 units of DNAase I from bovine pancreas (Boehringer Mannheim) in the presence of 20 units of RNAase inhibitor (Boehringer Mannheim) for 15 min at 37°C. DNAase I was inactivated by heating for 5 min at 65°C before cDNA preparation. cDNA was then synthesized from DNAase treated RNA in a 20-µl reverse transcription reaction mixture containing 100 ng of random hexamer primers (Pharmacia, Sollentuna, Sweden), 4 µl of buffer 5X, 2 µl of 0.1 M dithiothreitol (Gibco), 2 µl of dNTPs 5 mM (Boehringer), and 100 U of reverse transcriptase Superscript II (Gibco, UK). After 15 min at 42°C, cDNA was diluted to 1:5 with water then stored at – 20°C until use.

PCR amplification was performed on 5 µl of the RT product incubated with 1 U of *Taq* polymerase (ATGC, Noisy le grand, France) in a 25-µl reaction mixture containing 0.5 mM deoxynucleoside triphosphate, 2.5 mM magnesium chloride, 2.5 µl of 10 × *Taq* polymerase buffer from ATGC, 10 pmol of both specific and internal standard gene upstream and downstream primers to minimize tube-to-tube variations in amplification efficiency, and 1 µCi of [α-$^{32}$P]dCTP (Amersham, Les Ulis, France), which was always used within 5 days of the date of radiolabelling indicated by the manufacturer. The amplimer sequences for *MDR1* and β2 (Noonan et al, 1990), *PGK* (Ozcelik et al, 1995), *MRP* (Abbaszadegan et al, 1994) and *GSTp* (Morrow et al, 1989) are those previously published (Table 1).

© Cancer Research Campaign 1998

**Table 2** Characteristics of patients and samples

| Characteristics | n | % |
|---|---|---|
| Age (years) | | |
| < 50 | 23 | 31 |
| ≥ 50 | 51 | 69 |
| Tumour size (mm) | | |
| < 30 | 57 | 77 |
| ≥ 30 | 17 | 23 |
| Axillary nodal status | | |
| N– | 65 | 88 |
| N+ | 9 | 12 |
| Histological typing | | |
| Infiltrating ductal carcinomas | 63 | 85 |
| Infiltrating lobular carcinomas | 10 | 13 |
| Others | 1 | 2 |
| Histological grading (SBR) | | |
| I | 19 | 26 |
| II | 42 | 59 |
| III | 11 | 15 |
| Oestrogen receptor status | | |
| Negative | 14 | 28 |
| Positive | 35 | 72 |
| Progesterone receptor status | | |
| Negative | 16 | 33 |
| Positive | 33 | 67 |

*SBR, Scarff–Bloom and Richardson index. The cut-off value is 10 fmol $mg^{-1}$ protein for both oestrogen and progesterone receptors.

PCR was carried out in a thermal cycler (Perkin Elmer, Paris, France), and, after an initial denaturation at 95°C for 5 min, PCR consisted of 30 cycles, corresponding to the exponential range of the amplification reaction. The thermal profile was as follows: 60 s at 94°C, 60 s at 55°C and 90 s at 72°C. Negative control reactions, containing water instead of cDNA, were included in each experiment. Three microlitres of the radiolabelled PCR products were submitted to run on 8% polyacrylamide gel electrophoresis in a buffer containing 89 mM Tris-borate and 2 mM ethylenediamine tetraacetate disodium (EDTA, pH 8.3) at 50 V $cm^{-1}$. Gels were dried for 2 h at 80°C then exposed against X-O-Mat films (Eastman Kodak) for 6 h at – 80°C. Autoradiographs were densitometrically scanned on a Biorad densitometer (Biorad, Ivry, France) using Molecular Analyst software (Biorad). Specific gene expression was determined semiquantitatively by calculating the ratio of the densitometric values from specific genes expressed in relation to the internal standard.

### Determination of standard curves

For each gene investigated, a semiquantitative determination method of RT-PCR yields was developed using a standard curve established from three RT-PCR reactions from control cell lines assessed under identical reaction conditions. *MDR1* gene expression was determined by densitometry estimating the *MDR1*/β2 ratio from autoradiographs obtained following coamplification of *MDR1* and β2 cDNA from serial dilutions of drug-resistant KB 8-5 cells with drug-sensitive KB 3-1 cells.

**Figure 1** Semiquantitative RT-PCR determination of *MDR1*, *MRP* and *GSTp* gene expression in control cell lines. Standard curves (bottom) were established for *MDR1* (**A**), *MRP* (**B**) and *GSTp* (**C**) using serial dilution standard series of drug sensitive (KB 3.1, IGROV and MCF7S respectively) RNA and increasingly resistant cell line (KB 8.5 for *MDR1* and MCF7R for both *MRP* and *GSTp*) total RNA. Autoradiograms (top) of RT-PCR products (30 cycles) from MDR related genes and internal standards were densitometrically scanned for calculation of respective MDR-related gene/internal standard ratios. Inserts show the ranges of dilution for which amplification ratio and MDR-related gene mRNA showed a linear increment and were strictly proportional. The parametric Pearson correlation was used for data analysis

© Cancer Research Campaign 1998

*MRP/PGK* and *GSTp/PGK* standard curves were established by serial dilutions of MCF7R cells with either IGROV or MCF7S cells respectively.

The values of these ratios were independently assessed for respective control cell lines on six experiments performed under identical reaction conditions and the coefficient of variation was calculated for each one.

### Statistical analysis

Linear regression was used to model standard curves (with Pearson coefficient of correlation). Spearman rank order correlation analysis was performed to test all other continuous variables, namely correlation studies between the expression of the three genes. Mann–Whitney non-parametric tests were performed to compare continuous clinicopathological and laboratory variables. A nominal significance level of $\alpha' = 0.01$ was used for individual tests to guarantee an overall level of $\alpha = 0.05$ with ten stages.

### RESULTS

### Sample characteristics and flow cytometry

Table 2 summarizes the main characteristics of the samples: 23 (31%) patients were under 50 years of age, ranging from 21 to 49 years, and 51 (69%) were over 50 years old, ranging from 50 to 84 years with a mean of 42.2 ± 6.1 and 60.2 ± 8.2 respectively. The majority of the tumours were locally non advanced, as 57 (77%) were less than 3 cm in diameter (15.4 ± 6.1 mm) and 65 (88%) were node negative. All tumours were non-metastatic. All were invasive tumours: 63 (85%) were ductal cancers whereas ten (13%) were of lobular origin; only one was of medullary type and was not taken into account in the subsequent statistical analysis. The hormonal status was available on 49 samples for tissue receptor determination: 35 (72%) were classified as oestrogen receptor (ER)-positive, whereas 33 (67%) were considered to be progesterone receptor (PR)-positive.

Sixty four samples were evaluated for flow cytometry studies. Tumours containing a single cell population with a DNA index ranging between 0.9 and 1.1 were classified as diploid ($n$ = 34, 55%); those with an additional cell population with a DNA index beyond the limits of 0.9 and 1.1 were defined as aneuploid ($n$ = 28, 45%). None of the samples was exclusively composed of a single cell population with an aneuploid index.

### RT-PCR analysis

*Determination of standard curves*

Standard curves for RT-PCR analysis are shown on Figure 1.

The *MDR1* standard curve was obtained by submitting serial dilutions of total RNA from KB 8.5 cells mixed with total RNA extracted from KB 3.1 cells not expressing the *MDR1* gene to RT-PCR (Figure 1A). This curve was used to define a range of dilutions within which the *MDR1*/β2 ratio increased in a strictly linear fashion with the dilution factor. As shown more precisely on the inset, this range extended from 1/256 to 1/16 ($y = 9.4x + 0.03$, $r$ = 0.992). This result is consistent with that reported by Chevillard et al (1996) using the KBA1 cell line as *MDR1*-positive control. Under these conditions, with linear increment of the curve, the *MDR1*/β2 ratio increased in proportion to increasing concentrations of *MDR1* mRNA contained in the KB 8.5/KB 3.1 RNA mix and there was no competition for PCR between the two couples of primers. Finally, as reported in Table 3, the coefficient of variation calculated from six determinations of the *MDR1*/β2 ratio on KB 8.5 assessed under identical reaction conditions was low (8%), allowing us to compare tumour samples with KB 8.5 cells in subsequent rounds of PCR.

To establish the *MRP* standard curve, total RNA from the MRP-positive MCF7R cell line was diluted in RNA extracted from the low

**Table 3** RT-PCR determination in control cell lines (arbitrary units)

| | MDR1/β2 mean ± s.d. (CV %) | MRP/PGK mean ± s.d. (CV %) | GST p/PGK mean ± s.d. (CV %) |
|---|---|---|---|
| KB 3.1 | 0 (0%) | – | – |
| KB 8.5 | 0.836 ± 0.064 (8%) | – | – |
| MCF7S | – | – | 0 (0%) |
| MCF7R | – | 1.140 ± 0.051 (4.5%) | 0.784 ± 0.055 (7%) |
| IGROV | – | 0.243 ± 0.018 (7.4%) | – |

The coefficient of variation (CV) was calculated on six independent experiments.

**Figure 2** RT-PCR analysis of tumour samples. *MDR1*, *MRP* and *GSTp* mRNA content was expressed in relation to respective internal standards (β2 for *MDR1* and *PGK* for both *MRP* and *GSTp*). The results are expressed as the percentage of the ratio assessed in each set of experiment for positive control cell lines (KB 8.5 and MCR7R for *MDR1* and both *MRP* and *GSTp* respectively). Horizontal bars represent the mean values of the series

**Table 4** MDR phenotype and characterisics of tumours

| | Mean ± s.d. | | |
|---|---|---|---|
| **Characteristics** | **MDR1** | **MRP** | **GSTp** |
| Age (years)[a] | | | |
| < 50 | 19.2 ± 31.3 | 66.1 ± 26.1 | 85.1 ± 7.7 |
| ≥ 50 | 12.0 ± 18.1 | 57.3 ± 20.3 | 77.34 ± 31.0 |
| Tumour size (mm) | | | |
| < 30 | 14.08 ± 24.2 | 56.8 ± 20.7 | 77.8 ± 29.8 |
| ≥ 30 | 13.07 ± 20.5 | 70.1 ± 26.4 | 85.6 ± 43.6 |
| Axillary nodal status | | | |
| N– | 14.7 ± 24.1 | 59.2 ± 23.1 | 80.5 ± 33.1 |
| N+ | 10.9 ± 12.9 | 66.1 ± 17.2 | 74.7 ± 32.8 |
| Histological typing[b] | | | |
| Infiltrating ductal carcinomas | 14.4 ± 21.9 | 62.4 ± 23.2 | 80.8 ± 33.8 |
| Infiltrating lobular carcinomas | 14.9 ± 30.9 | 47.3 ± 9.8 | 73.9 ± 29.5 |
| Histological grading SBR[c] | | | |
| I | 13.9 ± 26.8 | 63.0 ± 23.9 | 79.6 ± 36.4 |
| II | 13.7 ± 17.8 | 62.2 ± 22.6 | 80.4 ± 29.7 |
| III | 18.9 ± 31.1 | 67.4 ± 26.4 | 91.3 ± 45.8 |
| Oestrogen receptor status | | | |
| Negative | 12.5 ± 14.6 | 51.8 ± 19.0 | 74.0 ± 44.8 |
| Positive | 11.0 ± 20.1 | 59.3 ± 19.6 | 84.5 ± 30.0 |
| Progesterone receptor status | | | |
| Negative | 12.5 ± 13.7 | 55.7 ± 19.3 | 78.0 ± 41.6 |
| Positive | 10.9 ± 20.7 | 57.9 ± 19.9 | 83.2 ± 31.4 |

[a]Age of patients; [b]other histological types were excluded; [c]SBR, Scarff–Bloom and Richardson index; Values are mean ± s.d. of mRNA levels ratios of *MDR1*, *MRP*, *GSTp* RT-PCR products expressed as a percentage of control cell lines. Mann–Witney tests were used for comparison of these variables. No significant statistical difference was observed. For SBR subgroups, expression of each MDR-related gene was compared between group I and II, I and III and II and III.

*MRP* expressing IGROV cell line and submitted to RT-PCR (Figure 1B). Under these conditions, the *MRP/PGK* ratio increased linearly for MCF7R/IGROV RNA dilutions ranging between 1/32 and 1/1 ($y = 0.85x + 0.26$, $r = 0.975$). This linear increment for low dilutions can be explained by the relatively low level of *MRP* expression by MCF7R and by the fact that IGROV cells were not totally negative for *MRP* expression (Table 3). As for *MDR1* determination, these RT-PCR procedures, designed to determine *MRP* expression, were highly reproducible on the MCF7R cell line with a coefficient of variation of 4.5% on six separate experiments (Table 3).

Similarly, to establish the *GSTp* standard curve, total RNA from the *GSTp*-positive MCF7R cell line was diluted in GSTp-negative MCF7S cells RNA and submitted to RT-PCR (Figure 1C). MCF7 was the first cell line described to overexpress *GSTp* when selected by doxorubicin and was subsequently used as a reference for *GSTp* expression (Batist et al,1986; Moscow et al, 1989). High dilutions, ranging between 1/512 and 1/16, were characterized by a high slope ($y = 4.8x + 0.003$, $r = 0.977$), whereas weak dilutions, ranging between 1/8 and 1/1, were characterized by a low slope ($y = 0.33x + 0.49$, $r = 0.98$). Nevertheless, it must be emphasized that, under these conditions, a plateau could never have been reached. The coefficient of variation for MCF7R was 7% when calculated on six *GSTp/PGK* ratio determinations (Table 3).

### *RT-PCR analysis of tumour samples*

Figure 2 shows the determination of *MDR1*, *MRP* and *GSTp* gene expression in the 74 samples. When compared with negative KB 3.1 and positive KB 8.5 control cell lines, 13 (17.6%) of the

**Figure 3** MDR related gene expression and DNA ploidy. Tumours were classified as diploid when the DNA index ranged between 0.9 and 1.1 ($n = 34$) and aneuploid when the DNA index was outside these values ($n = 28$). RT-PCR products of the three MDR-related genes are expressed as a percentage of control cell lines. The two groups of samples were compared using the Mann–Whitney non-parametric test; $P < 0.01$ was considered to be significant (*) (a nominal significance level of $\alpha' = 0.01$ was used for individual test to guarantee an overall level of $\alpha = 0.05$ with ten stages)

© Cancer Research Campaign 1998

**Figure 4** Correlation studies between *MDR1*, *MRP* and *GSTp* gene expression in breast tumours. Yields of RT-PCR products are expressed as a percentage of the MDR-related gene/internal standard compared with the positive control cell lines. For *MDR1* and *MRP* (**A**), *MDR1* and *GSTp* (**B**) and *MRP* and *GSTp* (**C**) expression data, Spearman rank order correlation analysis was used; $P < 0.05$ was considered to be significant samples definitely did not express the *MDR1* gene, whereas 61 (82.4%) were found to express *MDR1*: 45 (60.8%) of these samples showed a gene expression less than 20% of that of KB 8.5; 14 (18.9%) showed moderate expression, between 20 and 100%, whereas only two samples (2.7%) showed slightly higher expression than the control (101% and 104% of KB 8.5 respectively). If we correlate these values, expressed as a percentage of the positive control cell line, to the standard curve established by dilutions of the RNA control cell lines, 72 samples (97.3%) corresponded to the linear portion of the curve (dilution 1/256 to 1/16 of the KB 8.5 mRNA dilutions). This observation allowed us to validate our semiquantitative RT-PCR method when applied to samples expressing a relatively low level of *MDR1* mRNA, such as breast cancers.

For *MRP* expression, the values of *MRP/PGK* ratio ranged between 21% and 146% (mean ± s.d.: 60 ± 22.4). Again, only three tumours expressed *MRP/PGK* ratio at higher levels than the MCF7R control cell line (102, 117, and 146% of the MCF7R values respectively). Considering that the standard curve showed a linear increment for dilutions between 1/32 and 1/1, 70 of the 74 (94.6%) tumours corresponded to this portion of the curve. The majority of our samples were therefore able to be evaluated for *MRP* gene expression using this method.

Finally, concerning *GSTp* expression, the *GSTp/PGK* ratio ranged between 35% and 197% (mean ± s.d.: 79.8 ± 32.9) of the MCF7R control cell line. Twenty samples (27%) had a higher ratio than MCF7R. The distribution of these values on the standard curve indicated that 31 of the 74 tumours (41.9 %) corresponded to the higher slope of the curve (dilutions 1/512 to 1/8), whereas 23 of 74 (31.1%) corresponded to the second part of the curve (lower slope).

Table 4 reports the levels of expression of the three different genes in relation to the clinical and laboratory findings of patients and samples. As shown, none of the various subgroups expressed any of the MDR-related genes with a significant statistical difference. However, as shown on Figure 3, diploid tumours expressed *GSTp* at significantly higher levels than aneuploid tumours (93.6 ± 33.5 vs 70.9 ± 28.7% of controls, $P < 0.007$). In contrast, the proliferation state of the two types of tumours did not influence the expression of the three genes (data not shown).

Correlation studies between the expression of the three genes investigated also revealed a potentially important finding. To compare the *MDR1* levels to the levels of the other two drug resistance genes, we checked that a correlation close to 1:1 was observed between β2 and *PGK* levels in clinical samples (not shown). As shown on Figure 4, although *MDR1* and either *MRP* or *GSTp* gene expression did not seem to be correlated, our study revealed a significant positive correlation between *MRP* and *GSTp* expression when using Spearman correlation (rho = 0.47, $P < 0.0015$).

### DISCUSSION

The development of accurate and reliable tests to identify MDR determinants in clinical studies is now one of the major goals in the follow-up of cancer chemotherapy. Only a few of the many putative molecular mechanisms for clinical chemoresistance can be semiroutinely investigated. In addition to the P-gp mediated multidrug phenomenon, which has been extensively investigated in human pathology (Fojo et al, 1987; Pastan and Gottesman, 1987; Gottesman et al, 1989; Noonan et al, 1990; Weinstein et al, 1990; Areci et al, 1993 and cited in Gottesman and Pastan, 1993), other mechanisms have been more recently identified; some, such as MRP-mediated multidrug resistance, have not been well investigated, whereas others, such as GSTp, have not been clearly identified as directly involved in MDR and need to be investigated in conjunction with other factors.

Our study reports reliable experimental RT-PCR procedures that are able to simultaneously measure mRNA levels of three MDR-related genes. A variety of methods using either quantitative or semiquantitative RT-PCR have been used to determine relative initial target mRNA in samples. However, in all of these methods undefined variations in amplification efficiency complicate the interpretation of results and, in an attempt to correct for tube-to-tube variations in amplification efficiency, most investigators use internal amplification standards, either gene transcript normally present in the sample, or an exogenous fragment added to the amplification reaction. In this study, as in many other studies, particularly concerning human tumour tissue samples (Noonan, 1990; Horikoshi, 1992; Chevillard, 1996), we chose to use endogenous standards. One of the greatest advantages of using the expression of an endogenous sequence as an internal standard is that the reference mRNA and target mRNA are usually processed

© Cancer Research Campaign 1998

together throughout the experiment, i.e. from RNA extraction until PCR amplification. This tends to minimize differences in RNA yield between samples, an important advantage, particularly for analysis of small tissue samples. In addition, if the entire population of RNA is converted to cDNA, the overall efficiency of cDNA synthesis is somewhat normalized. Our assays were also performed in agreement with the consensus recommendations recently proposed by the workshop on *MDR1* evaluation (Beck et al, 1996): to work on a dominant tumour cell population in the exponential range of the amplification reaction; to choose optimal internal standards; to avoid any competition between primer pairs in multiplex reactions; and finally to establish PCR assays with negative controls as well as standard series of drug-sensitive and increasingly resistant cell lines to establish a titration of the sequences to be amplified and to be sure that the target/standard PCR products ratio increases linearly with the initial concentration of the target sequence during the exponential phase of amplification of the two sequences. Concerning this last point, the standard curves determined in the present work for the three genes investigated, demonstrate that the conditions used here are validated for the relatively low mRNA levels measured in our series of breast cancer samples.

The choice of relevant control cell lines is particularly important to validate such a method. Both parental and drug-resistant KB and MCF7 cell lines are well referenced control cell lines to study *MDR1* and *GSTp* gene expression (Gottesman, 1993; Moscow et al, 1989). The MCF7R cell line expresses MRP at a relatively lower level than other classical positive control cell lines, such as H69AR (Cole, 1992) and GLC4 /ADR (Zijlstra et al, 1987; Zaman et al, 1993). Nevertheless, the use of this cell line as positive control for MRP in our study on breast cancer can be justified, as it is derived from human breast cancer and also because there is growing evidence that MRP can act as a membranous glutathione conjugate carrier (see below). We, therefore, preferred to use the same positive control cell line for both *MRP* and *GSTpi* expression. The IGROV cell line was chosen as low expressing control cell line because, to our knowledge, few other cell lines have been shown to express *MRP* at low levels when using ultrasensitive detection methods such as RNAase protection assay or RT-PCR except the parental H69 cell line as reported by Cole et al (1992). Indeed, like the GLC4 cell line (Nooter et al, 1995), the IGROV cell line is weakly positive for MRP expression. Furthermore, the IGROV cell line expressed the *PGK* internal standard gene at an identical level to the MCF7R-positive control cell line.

Concerning the choice of the internal standards β2 and *PGK*, β2 is the gene recommended for calibrating *MDR1* in the recent consensus recommendations. However as MCF7R failed to express the β2 gene, we therefore had to choose another gene constitutively expressed in breast, namely *PGK*, to calibrate *MRP* and *GSTpi* expression (Ozcelik et al, 1995).

From a molecular point of view, the mechanisms of MDR are often opportunistic in their manipulation and modification of normal pathways of cellular homeostasis. Consequently, it would be interesting to evaluate whether a particular subgroup of untreated breast carcinomas could be isolated before chemotherapy on the basis of MDR phenotype.

We showed that *MDR1* expression was relatively weak in the overall panel of samples and appeared independent of both clinicopathological and laboratory features. The present study shows that *MDR1* mRNA quantitation can be considered as an independent drug resistance marker in untreated breast cancer when compared with the expression of other main MDR-related genes. One of the present questions is whether the assessment of mRNA by RT-PCR of certain drug resistance markers is relevant to clinical end points of treatment when compared with protein. Charpin et al (1994) evidenced a good correlation between quantitative immunocytochemical assay for P-gp and PCR analysis of *MDR1* expression in frozen untreated breast carcinomas. Alvarez et al (1995) described a drug resistance profile when they quantitated *MDR1* expression by RT-PCR in cell lines. Overexpression of either the *MDR1* gene or its P-gp product has been frequently reported in breast cancer and has often been linked to either in vitro resistance phenomenon or clinical resistance, namely to anthracyclines (Fojo et al, 1987; Merckel et al, 1989; Salmon et al, 1989; Schneider et al, 1989; Keith et al, 1990; Ro et al, 1990; Wishart et al, 1990; Sanfilipo et al, 1991; Verelle et al, 1991; Wallner et al, 1991; Hennequin et al 1993; Chevillard et al, 1996). In addition, the presence of increased levels of P-gp in several types of tumours has been correlated with short progression-free survival and overall survival (van Kalken et al, 1991). Gregorcyk et al (1996) demonstrated a greater risk of recurrence at 5 years in P-gp overexpressing untreated breast carcinomas. Finally, P-gp overexpression has been correlated with c-*erb*B-2 expression, which is known to be another poor prognostic marker in breast carcinomas (Brotherick et al, 1996).

*MRP* is ubiquitously expressed in normal human tissue (Zaman et al, 1993; Kruh et al, 1995; Nooter et al, 1995), but few data are available concerning *MRP* expression in human cancers. Kruh et al (1995) detected transcripts in all of nine breast cancer cell lines. Using a RNAase protection assay, Nooter et al (1995) classified breast carcinomas as low *MRP*-expressing tumours. In the present study, we confirm that almost all samples tested expressed relatively low levels of *MRP* mRNA. Using the MRP-specific monoclonal antibody MRPr1 Flens et al (1996), Nooter et al (1997) recently reported the first study showing a higher MRP expression in the operable primary tumour of recurrent breast cancers treated with first-line systemic chemotherapy. The present work provides additional evidence concerning a possible relationship with GSTpi in breast carcinoma (see below).

Our results concerning *GSTp* mRNA levels are in accordance with those published by Moscow et al (1988). However, as reported by Shea et al (1990) and Peters et al (1993), we did not find any relationship between GSTp expression and hormone receptor status. Such a result is at variance with previous reports (Moscow et al, 1988; Gilbert et al, 1993). In addition, we also showed that diploid tumours exhibited higher *GSTp* mRNA levels than aneuploid tumours. Although GSTp has not been unambiguously directly involved in MDR (Moscow et al, 1988; Tew, 1994 and cited in Morrow et al, 1993), its value as a prognostic marker has to be appraised. Indeed, an increased GSTp expression has been suggested to be predictive of early recurrence and death in node-negative breast cancer (Gilbert et al, 1993; Silverstini et al, 1997), particularly when not treated by adjuvant radiotherapy (Silverstini et al, 1997).

Finally, one of the main findings of our study is the suggested relationship between MRP and GSTp expression. There is convincing evidence that the transport of glutathione conjugates (GS-X) might be mediated by the membranous MRP, GS-X pump (Müller et al, 1994; Zaman et al, 1995). In addition, it has been suggested that the MRP/GS-X pump might transport the metabolic

© Cancer Research Campaign 1998

derivatives of doxorubicin, but not the native drug (Cole et al,1994). Zaman et al (1995) have provided important evidence that cellular GSH is a critical factor for the export of doxorubicin by the MRP/GS-X pump and Ishikawa et al (1995) proposed a scheme indicating that both MRP/GS-X pump and metabolic enzyme systems including GST, as well as GSH, could be critically involved in the mechanisms underlying doxorubicin resistance. The correlation between MRP and GSTp expression suggests that genes coding for conjugation enzymes of toxic compounds to GSH, such as GSTp, as well as MRP/GS-X pump might be co-regulated during the development of breast tumours before any chemotherapy and could subsequently be involved in clinical chemoresistance. Resistance to many anti-cancer drugs has been linked to increased cellular levels of GSH and glutathione-S-transferases (Tew, 1994) and, although conjugates of anthracyclines and vinca alkaloids with GSH have not been described, this finding is in favour of older experiments in which resistance to anthracyclines was found to be correlated with increased levels of cellular GSH, GSH synthesis or glutathione-S-transferases (cited in Morrow, 1993 and Tew, 1994). Such a hypothesis is in accordance with the recent report (Kuo et al, 1996) showing, in human colorectal cancers, a frequent coordinated overexpression of the *MRP* gene and the g-glutamylcysteine synthetase (g-*GCS*) gene, an enzyme involved in GSH synthesis.

In conclusion, accurate and reliable quantitation of different types of MDR-related gene expression by fine and sensitive methods such as RT-PCR presented in this paper, could provide a powerful tool to easily investigate possible interrelationships between these genes on a single tumour sample.The purpose of this methodological paper was not to provide data on treatment outcome of patients, but the clinical relevance of our data now needs to be investigated by prospective clinical correlative studies, namely by sequential semi-quantitative determination of different MDR-related gene expression in terms of prediction of response to chemotherapy, design of studies aimed at reversal of drug resistance and also in terms of the overall prognosis of breast carcinomas.

### ACKNOWLEDGEMENTS

This work was supported in part by the ACTT (Amis du Centre des Tumeurs de Tenon – Paris). We thank M Gottsman, J Robert, and J Benard for providing control cell lines, M Lestrat for help in collecting data and V Gerber for editorial assistance.

### REFERENCES

Abbaszadegan MR, Futcher BW, Klimecki WT, List A and Dalton W (1994) Analysis of multidrug resistance associated protein (MRP) messenger RNA in normal and malignant hematopoietic cells. *Cancer Res* **54**: 4676–4679

Akiyama S, Fojo A, Hanover JA, Pastan I and Gottesman MM (1985) Isolation and genetic characterization of human KB cell lines resistant to multiple drugs. *Som Cell Mol Genet* **11**: 117–126

Almquist KC, Loe DW, Hipfner DR, Mackie JE, Cole SPC and Deeley RG (1995) Characterization of the Mr 190,000 Multidrug Resistance Protein (MRP) in drug-selected and transfected human tumor cells. *Cancer Res* **55**: 102–110

Alvarez M, Paull K, Monks A, Hose C, Lee JS, Weinstein J, Grever M, Bates S and Fojo T (1995) Generation of a drug resistance profile by quantitation of mdr-1/P-glycoprotein in the cell lines of the National Cancer Institute Anticancer Drug Screen. *J Clin Invest* **95**: 2205–2214

Areci RJ (1993) Clinical significance of P-glycoprotein in multidrug resistance malignancies. *Blood* **81**: 2215–2222

Batist G, Tulpule A, Sinha BK, Katki AG, Myers CE and Cowan K.H (1986) Overexpression of a novel anionic glutathione transferase in multidrug-resistant human breast cancer cells. *J Biol Chem* **261**: 15544–15549

Beck WT, Grogan TM, Willman CT, Cordon-Cardo C, Parham DM, Kuttesch JF, Andreeff M, Bates SE, Berard CW, Boyett JM, Brophy NA, Broxterman HJ, Chan HSL, Dalton WS, Dietel M, Fojo AT, Gascoyne RD, Head D, Houghton PJ, Srivastava DK, Lehnert M, Leith CP, Paietta E, Pavelic ZP, Rimsza L, Roninson IB, Sikic BI, Twentyman PR, Warnke R and Weinstein R (1996) Methods to detect P-glycoprotein-associated multidrug resistance in patients' tumors: consensus recommendations. *Cancer Res* **56**: 3010–3020

Bénard JD, Silva JD, Blois MC, Boyer P, Duvillard P, Chiric E and Riou G (1985) Characterization of human ovarian adenocarcinoma line, IGROV1, in tissue culture and in nude mice. *Cancer Res* **45**: 4970–4979

Breuninger LM, Paul S, Gaughan K, Miki T, Chan A, Aaranson SA and Kruh GD (1995) Expression of Multidrug Resistance-associated protein in NIH/3T3 cells confers multidrug resistance associated with increased drug efflux and altered intracellular drug distribution. *Cancer Res* **55**: 5342–5347

Brotherick I, Shenton BK, Egan M, Cunliffe WE, Browell DA, Lunt LG, Young JR, Higgs MJ (1996) Examination of multidrug resistance in cell lines and primary breast tumours by flow cytometry. *Eur J Cancer* **32A**: 2334–2341

Cole SPC, Bhardwaj G, Gerlach JH, Mackie JE, Grant CE, Almquist KC, Stewart AJ, Kurz EU, Duncan AMV and Deeley RG (1992) Overexpression of a transporter gene in a multidrug-resistant cell line. *Science* **258**: 1650–1654

Cole SPC, Sparks KE, Fraser K, Loe DW, Grant CE, Wilson GM and Deeley RG (1994) Pharmacological characterization of multi-drug resistant MRP-transfected human tumor cells. *Cancer Res* **54**: 5902–5910

Coste A, Rateau JG, Roudot-Thoraval F, Chapelin C, Gilain L, Poron F, Peynegre R, Bernaudin JF and Escudier E (1996) Increased epithelial cell proliferation in nasal polyps. *Arch Otolaryngol Head Neck Surg* **122**: 432–436

Charpin C, Vielh P, Duffaud F. Devictor B, Andrac L, Lavaut MN, Alasia C, Horschowski N and Piana L (1994) Quantitative immunocytochemical assays of P-glycoprotein in breast carcinomas: correlation to messenger RNA expression and to immunohistochemical prognostic indicators. *J Natl Cancer Inst* **86**: 1539–1545

Chevillard S, Pouillart P, Beldjord C, Asselain B, Beuzeboc P, Magdalena H and Vielh P (1996) Sequential assessment of multidrug resistance phenotype and measurement of S-Phase fraction as predictive markers of breast cancer response to neoadjuvant chemotherapy. *Cancer* **77**: 292–300

Endicott JA and Ling V (1989) The biochemistry of P-glycoprotein-mediated multidrug resistance. *Annu Rev Biochem* **58**: 137–171

Flens MJ, Izquierdo MA, Scheffer GL, Fritz JM, Meijer CJL, Scheper RJ and Zaman GJR (1994) Immunochemical detection of the multidrug resistance associated protein MRP in human multidrug resistant tumor cells by monoclonal antibodies. *Cancer Res* **54**: 4557–4563

Flens MJ, Zaman GJ, van der Valk P, Izquierdo MA, Schroeijers AB, Scheffer GL, van der Groep P, de Haas M, Meijer CJ and Scheper RJ (1996) Tissue distribution of the multidrug resistance protein. *Am J Pathol* **148**: 1237–1247

Fojo AT, Ueda K, Slamon DJ, Poplack DG, Gottesman MM and Pastan I (1987) Expression of a multidrug-resistance gene in human tumors and tissues. *Proc Natl Acad Sci USA* **84**: 265–269

Gilbert L, Elwood LJ, Merino M, Masood S, Barnes R, Steinberg SM, Lazarous DF, Pierce L, D'Angelo T, Moscow JA, Townsend AJ and Cowan KH (1993) A pilot study of pi-class glutathione S-transferase expression in breast cancer: correlation with estrogen receptor expression and prognosis in node-negative breast cancer. *J Clin Oncol* **11**: 49–58

Gottesman MM and Pastan I (1993) Biochemistry of multidrug resistance mediated by the multidrug transporter. *Annu Rev Biochem* **62**: 385–427

Gottesman MM, Goldstein LJ, Bruggemann E, Currier SJ, Galski H, Cardarelli C, Thiebaut F, Willingham MC and Pastan I (1989) Molecular diagnosis of multidrug resistance. *Cancer Cells* **7**: 75–80

Gregorcyk S, Kang Y, Brandt D, Kolm P, Singer G and Perry RP (1996) P-Glycoprotein expression as a predictor of breast cancer recurrence. *Ann Surg Oncol* **3**: 8–14

Hennequin E, Delvincourt C, Pourny C, Jardillier JC (1993) Expression of mdr1 gene in human breast primary tumors and metastases. *Breast Cancer Res Treat* **26**: 267–274

Horikoshi T, Danenberg KD, Stadlbauer THW, Volkenandt M, Shea LCC, Aigner K, Gustavsson B, Leichman L, Frösing R, Ray M, Gibson NW, Spears CP and Danenberg PV (1992) Quantitation of thymidylate synthase, dihydrofolate reductase, and DT-diaphoroase gene expression in human tumors using the polymerase chain reaction. *Cancer Res* **52**: 108–116

Ishikawa T, Akimaru K, Kuo MT, Priebe W and Susuki M (1995) How does the MRP/GS-X pump export doxorubicin? *J Natl Cancer Inst* **87**: 1639–1640

Jedlitschky G, Leier I, Buchholz U, Center M and Keppler D (1994) ATP-dependent transport of glutathione S-conjugates by the multidrug resistance-associated protein. *Cancer Res* **54**: 4833–4836

Keith WN, Stallard S and Brown R (1990) Expression of mdr1 and GSTpi in human breast tumours: comparison to in vitro chemosensitivity. *Br J Cancer* **61**: 712–716

Kuo MT, Bao J, Curley SA, Ikeguchi M, Johnston DA and Ishikawa T (1996) Frequent coordinated overexpression of the MRP/GS-X pump and g-glutamylcysteine synthetase genes in human colorectal cancers. *Cancer Res* **56**: 3642–3644

Kruh GD, Gaughan KT, Godwin A and Chan A (1995) Expression pattern of MRP in human tissues and adult solid tumor cell lines. *J Natl Cancer Inst* **87**: 1256–1258

Merkel DE, Fuqua SAW, Tandon AK, Hill SM, Buzdar AU and M Guire WL (1989) Electrophoretic analysis of 248 clinical breast cancer specimens for P-glycoprotein overexpression or gene amplification. *J Clin Oncol* **7**: 1129–1136

Morrow SC and Cowan KH (1993) Antineoplastic drug resistance and breast cancer. *Ann NY Acad Sci* **698**: 289–312

Morrow CS, Cowan KH and Goldsmith ME (1989) Structure of the human genomic glutathione S-transferase-p gene. *Gene* **75**: 3–11

Moscow JA, Townsend AJ, Goldsmith ME, Whang-Peng J, Vickers PJ, Poisson R, Legault-Poisson S, Myers CE and Cowan KE (1988) Isolation of the human anionic glutathione S-transferase cDNA and the relation of its gene expression to estrogen-receptor content in primary breast cancer. *Proc Natl Acad Sci USA* **85**: 6518–6522

Moscow JA, Fairchild CR, Madden MJ, Random DT, Wieand HS, O'Brien EE, Poplack DG, Cossman J, Myers CE and Cowan KH (1989) Expression of anionic glutathione S transferase and P glycoprotein genes in human tissues and tumors. *Cancer Res* **49**: 1422–1428

Müller M, Meijer C, Zaman GJR, Borst P, Scheper RJ, Mulder NH, D Vries EGE and Jansen PLM (1994) Overexpression of the gene encoding the multidrug resistance associated protein results in increased ATP-dependent glutathione S-conjugate transport. *Proc Natl Acad Sci USA* **91**: 13033–13037

Noonan KE, Beck C, Holzmayer TA, Chin JE, Wunder JS, Andrulis IL, Gazdar AF, Willman CL, Griffith B, V Hoff DD and Roninson IB (1990) Quantitative analysis of MDR1 (multidrug resistance) gene expression in human tumors by polymerase chain reaction. *Proc Natl Acad Sci USA* **87**: 7160–7164

Nooter K, Westerman AM, Flens MJ, Zaman GJR, Scheper RJ, V Wingerden KE, Burger H, Oostrum R, Boersma T, Sonneveld P, Gratama JW, Kok T, Eggermont AMM, Bosman FT and Stoter G (1995) Expression of the multidrug resistance-associated Protein (MRP) gene in human cancers. *Clin Cancer Res* **1**: 1301–1310

Nooter K, Brutel de la Rivière G, Klijn J, Stoter G and Foekens J (1997) Multidrug resistance protein in recurrent breast cancer. *Lancet* **349**: 1885–1886

Ozcelik H, Mousses S and Andrulis IL (1995) Low levels of expression of an inhibitor of cyclin-dependent kinases (CIP1/WAF1) in primary breast carcinomas with P53 mutations. *Clin Cancer Res* **1**: 907–912

Pastan I and Gottesman M (1987) Multiple-drug resistance in human cancer. *N Engl J Med* **316**: 1388–1393

Peters WH, Roelofs HM, van Putten WL, Jansen JB, Klijn JG and Foekens JA (1993) Response to adjuvant chemotherapy in primary breast cancer: no correlation with expression of glutathione S-transferases. *Br J Cancer* **68**: 86–92

Ro J, Sahin A, Ro JY, Fritsche H, Hortobagyi G and Blick M (1990) Immunohistochemical analysis of P-glycoprotein expression correlated with chemotherapy resistance in locally advanced breast cancer. *Hum Pathol* **21**: 787–791

Salmon SE, Grogan TM, Miller T, Scheper R and Dalton WS (1989) Prediction of doxorubicin resistance in vitro in myeloma, lymphoma and breast cancer by P-glycoprotein staining. *J Natl Cancer Inst* **81**: 696–701

Sanfilippo O, Ronchi E, DeMarco C, DiFronzo G. and Silvestrini R (1991) Expression of P-glycoprotein in breast cancer tissue and in vitro resistance to doxorubicin and vincristine. *Eur J Cancer* **27**: 155–158

Schneider WN, Bak M, Efferth TH, Kaufmann M, Marten J and Volm M (1989) P-glycoprotein expression in treated and untreated human breast cancer. *Br J Cancer* **60**: 815–818

Shea TC, Clafin G, Comstock KE, Sanderson BJS, Burstein NA, Keenan EJ, Mannervick B and Henner WD (1990) Glutathione transferase activity and isoenzyme composition in primary human breast cancers. *Cancer Res* **50**: 6848–6853

Silvestrini R, Veneroni S, Benini E, Daidone MG, Luisi A, Leutner M, Maucione A, Kenda R, Zucali R and Veronesi U (1997) Expression of p53 glutathione S-transferase-pi, and Bcl-2 proteins and benefit from adjuvant radiotherapy in breast cancer. *J Natl Cancer Inst* **89**: 639–645

Simon SM and Schindler M (1994) Cell biological mechanisms of multidrug resistance in tumors. *Proc Natl Acad Sci USA* **91**: 3497–3504

Slovak ML, Ho JP, Bhardwaj G, Kurz EU, Deeley RG and Cole SPC (1993) Localization of a novel multidrug resistance-associated gene in the HT1080/DR4 and H69AR human tumor cell lines. *Cancer Res* **53**: 3221–3225

Soule HD, Vazquez A, Long A, Albert S and Brennan M (1973) Human cell line from a pleural effusion derived from a breast carcinoma. *J Natl Cancer Inst* **51**: 1409–1413

Tew KD (1994) Glutathione-associated enzymes in anticancer drug resistance. *Cancer Res* **54**: 4313–4320

van Kalken CK, Pinedo HM and Giaccone G (1991) Multidrug resistance from the clinical point of view. *Eur J Cancer* **27**: 1481–1486

Verrelle P, Messonier F, Fonck Y, Feillel V, Dionet C, Kwiatkoswski F, Plagne R and Chassagne J (1991) Clinical relevance of immunohistochemical detection of multidrug resistance P-glycoprotein in breast carcinoma. *J Natl Cancer Inst* **83**: 111–116

Wallner J, Despich D, Hopfner M, Haider K, Spona J, Ludwig H and Pirker R (1991) MDR1 gene expression and prognostic factors in primary breast carcinomas. *Eur J Cancer* **27**: 1352–1355

Weinstein RS, Kuszak JR, Kluskens LF and Coon JS (1990) P-glycoproteins in pathology: the multidrug resistance gene family in humans. *Human Pathol* **21**: 34–48

Wishart GC, Plumb JA, Going JJ, M Nicol AM, M Ardle CS, Tsuruo P and Kaye SB (1990) P-glycoprotein expression in primary breast cancer detected by immunocytochemistry with two monoclonal antibodies. *Br J Cancer* **62**: 758–761

Zaman GJR, Versantvoort CHM, Smit JJM, Eijdems EWHM, d Haas M, Smith AJ, Broxterman HJ, Mulder NH, d Vries EGE, Baas F and Borst P (1993) Analysis of the expression of MRP, the gene for a new putative transmembrane drug transporter, in human multidrug resistant lung cancer cell lines. *Cancer Res* **53**: 1747–1750

Zaman GJR, Flens MJ, V Leusden MR, D Haas M, Mulder NH, Lankelma J, Pinedo J, Scheper HM, Baas RJ, Broxterman HJ and Borst P (1994) The human multidrug resistance-associated protein MRP is a plasma membrane drug-efflux pump. *Proc Natl Acad Sci USA* **91**: 8822–8826

Zaman GJR, Lankelma J, V Tellingen O, Beijnen J, Dekker H, Paulusma C, Elferink RPJO, Baas RJ and Borst P (1995) Role of glutathione in the export of compounds from cells by the multidrug-resistance-associated protein. *Proc Natl Acad Sci USA* **92**: 7690–7694

Zijlstra JG, D Vries EGE and Mulder NH (1987) Multifactorial drug resistance in an adriamycin-resistant human small cell lung carcinoma cell line. *Cancer Res* **47**: 1780–1784

© Cancer Research Campaign 1998


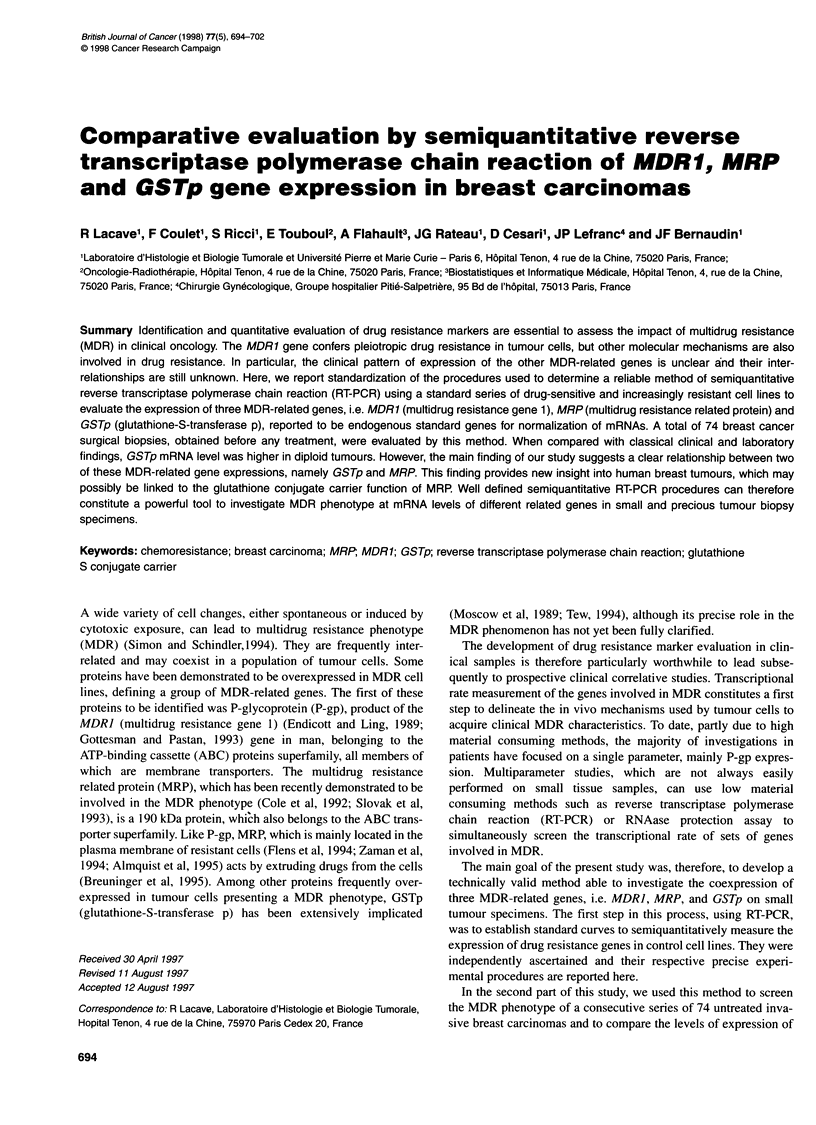

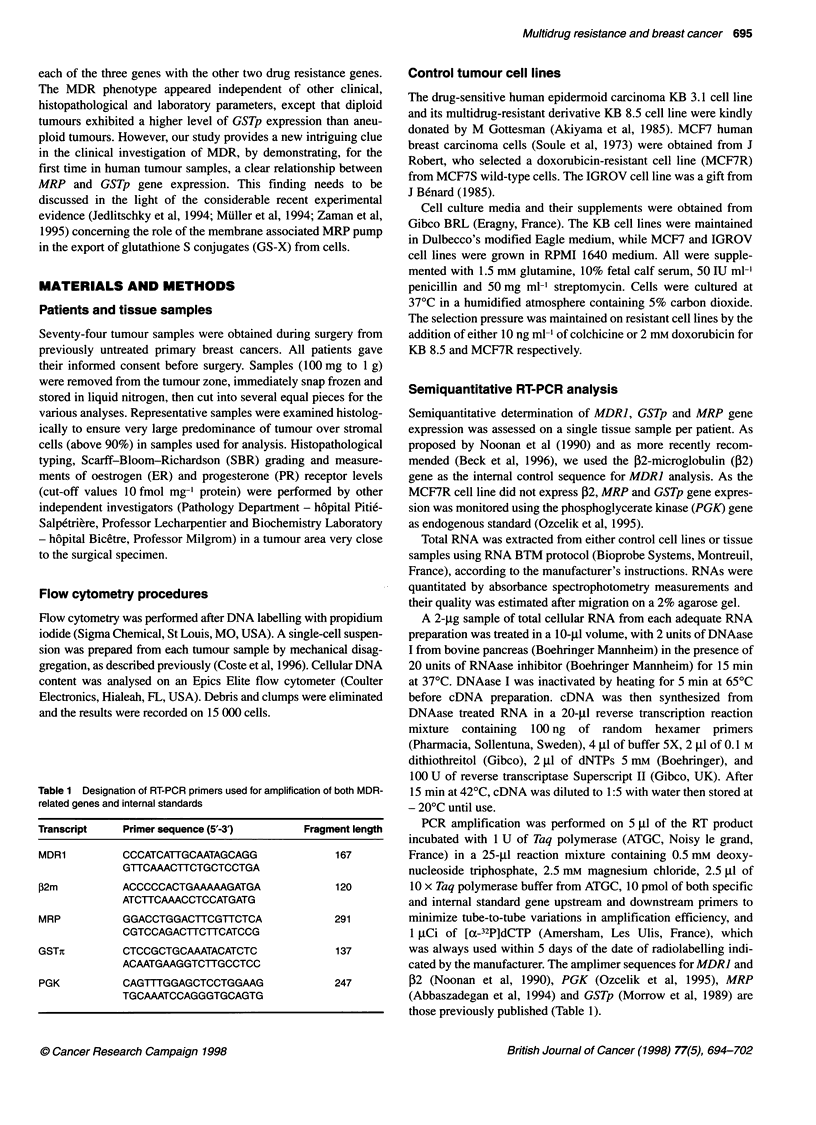

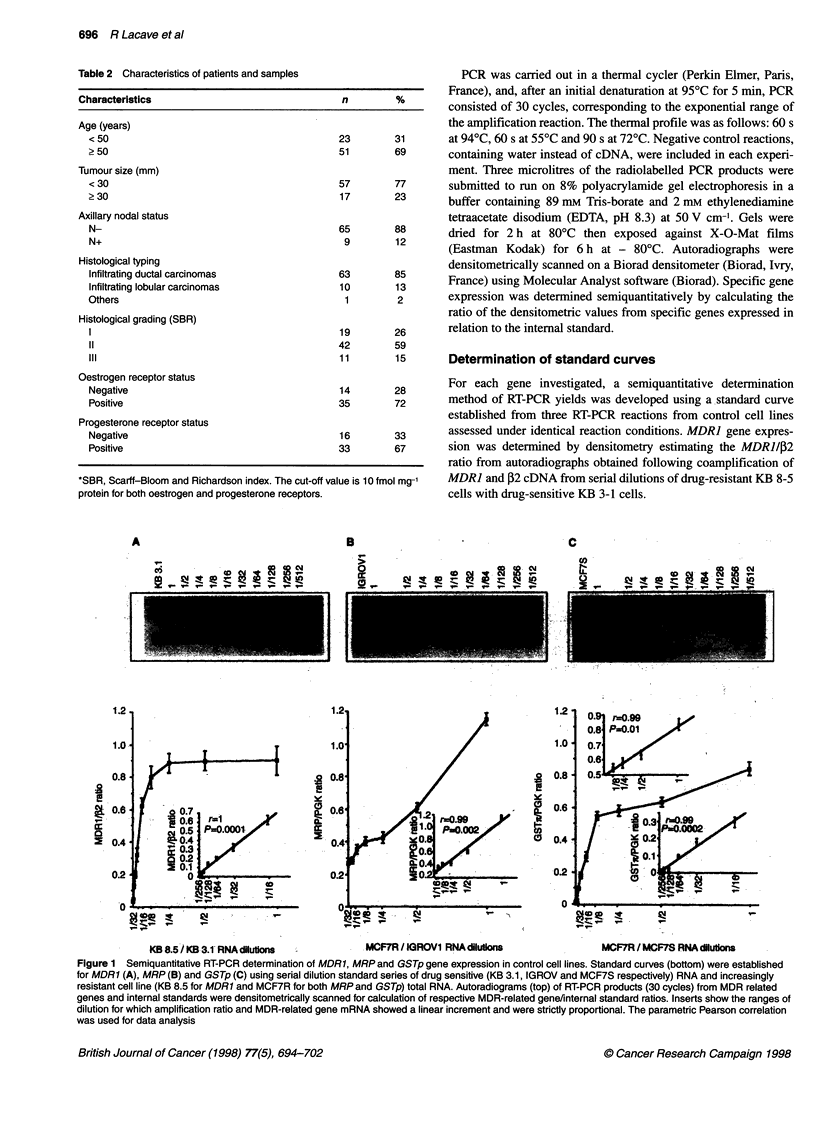

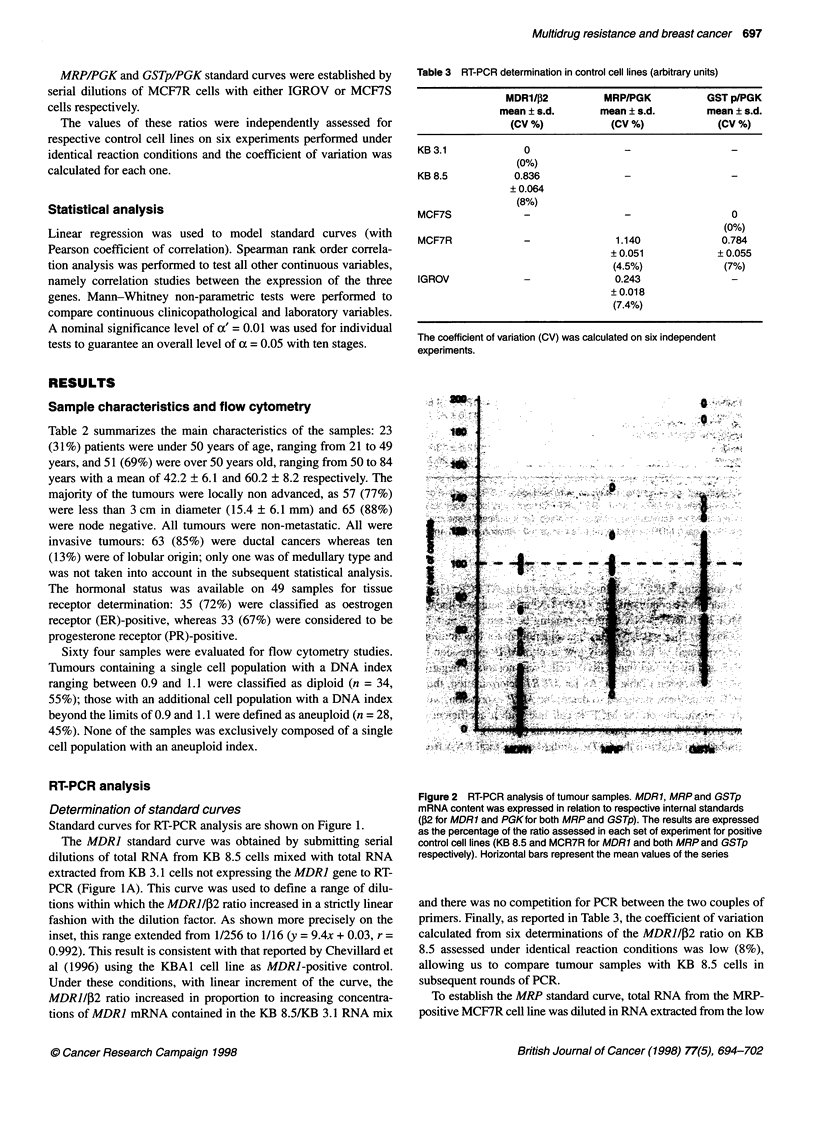

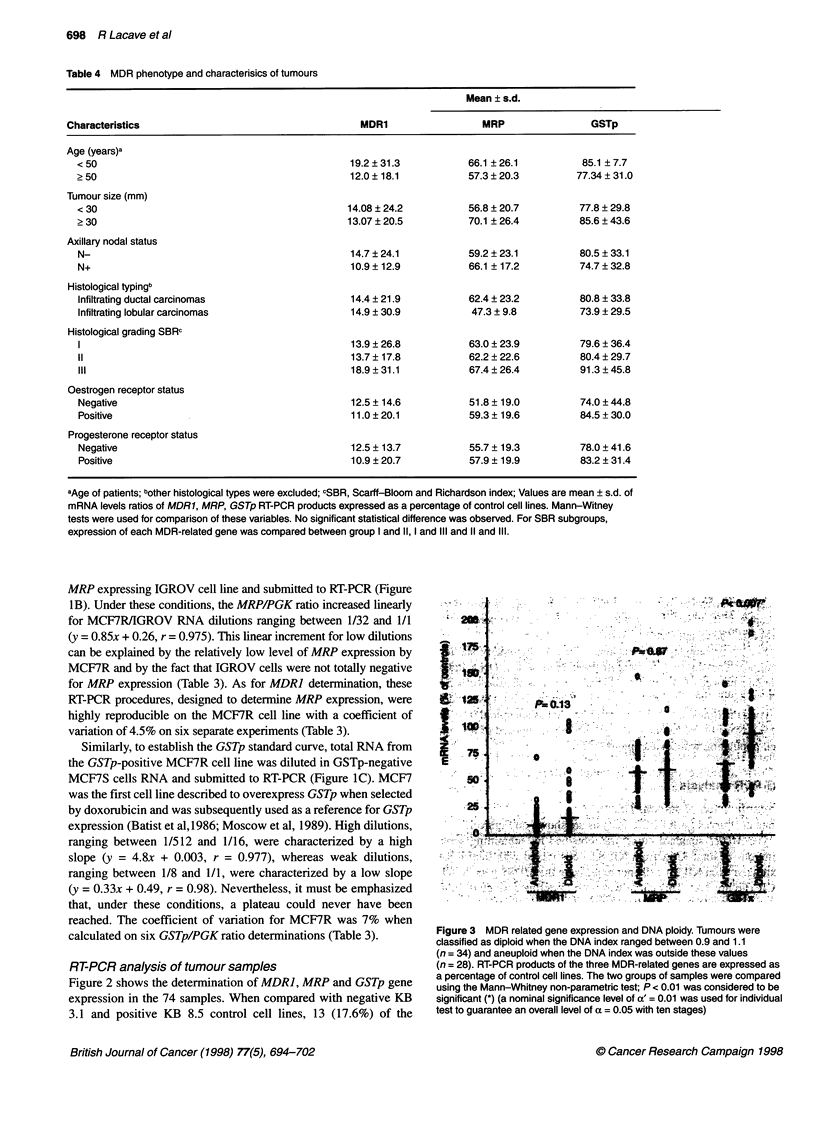

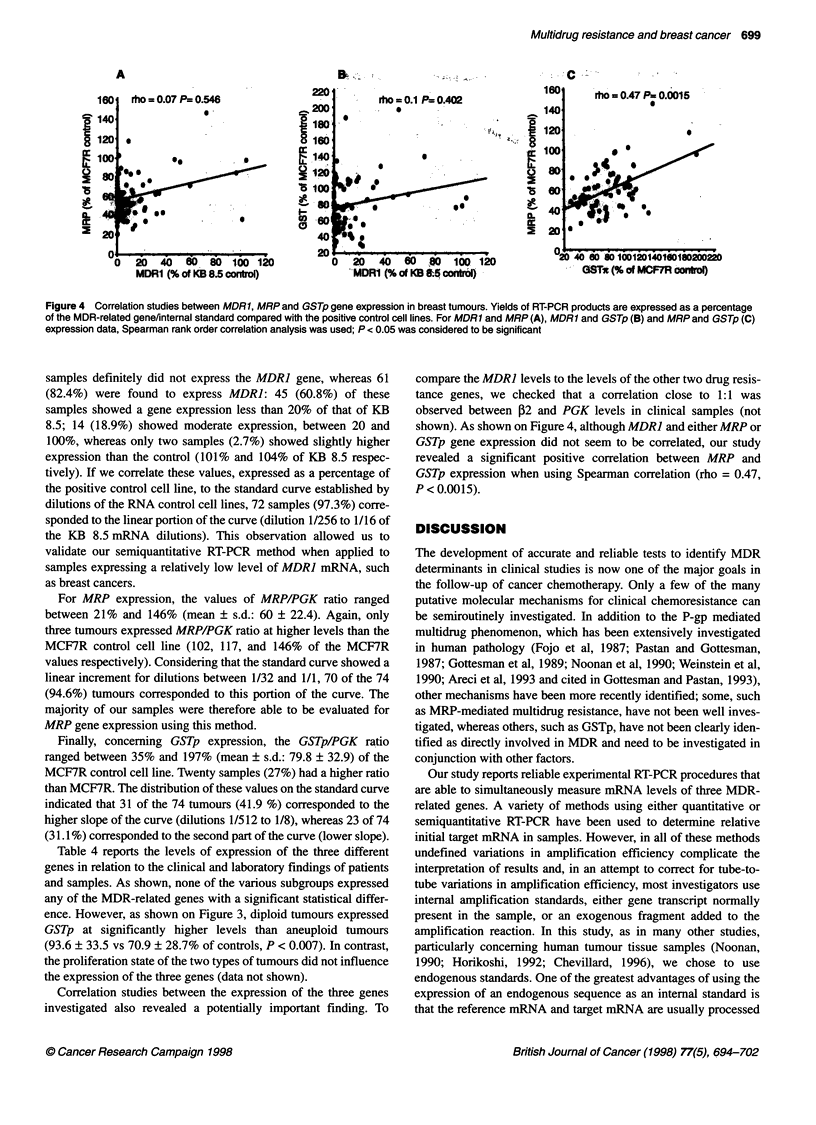

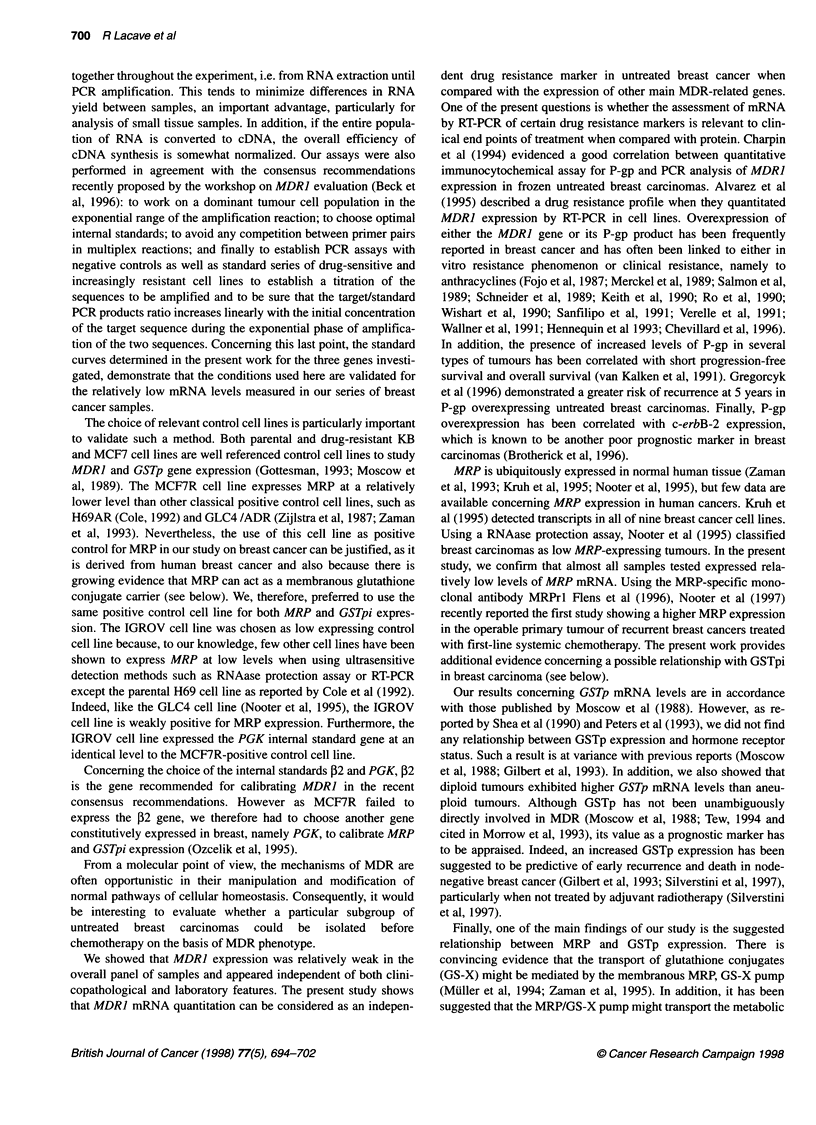

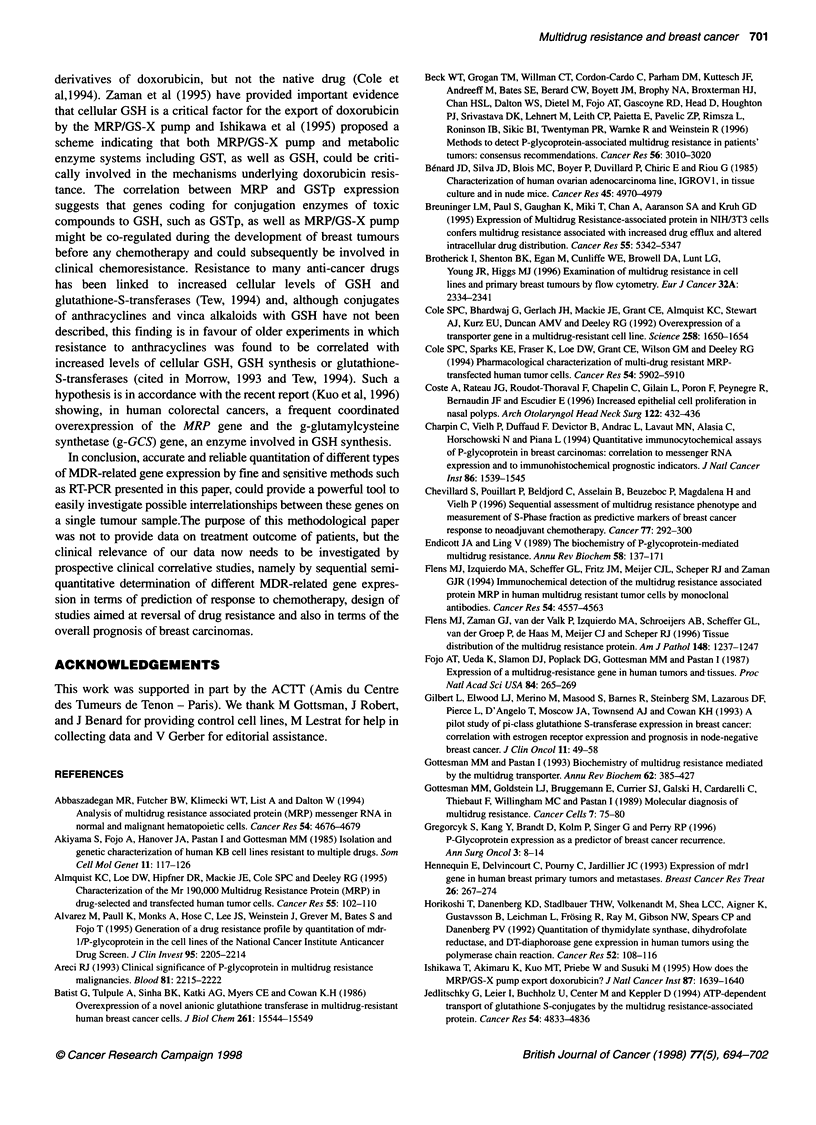

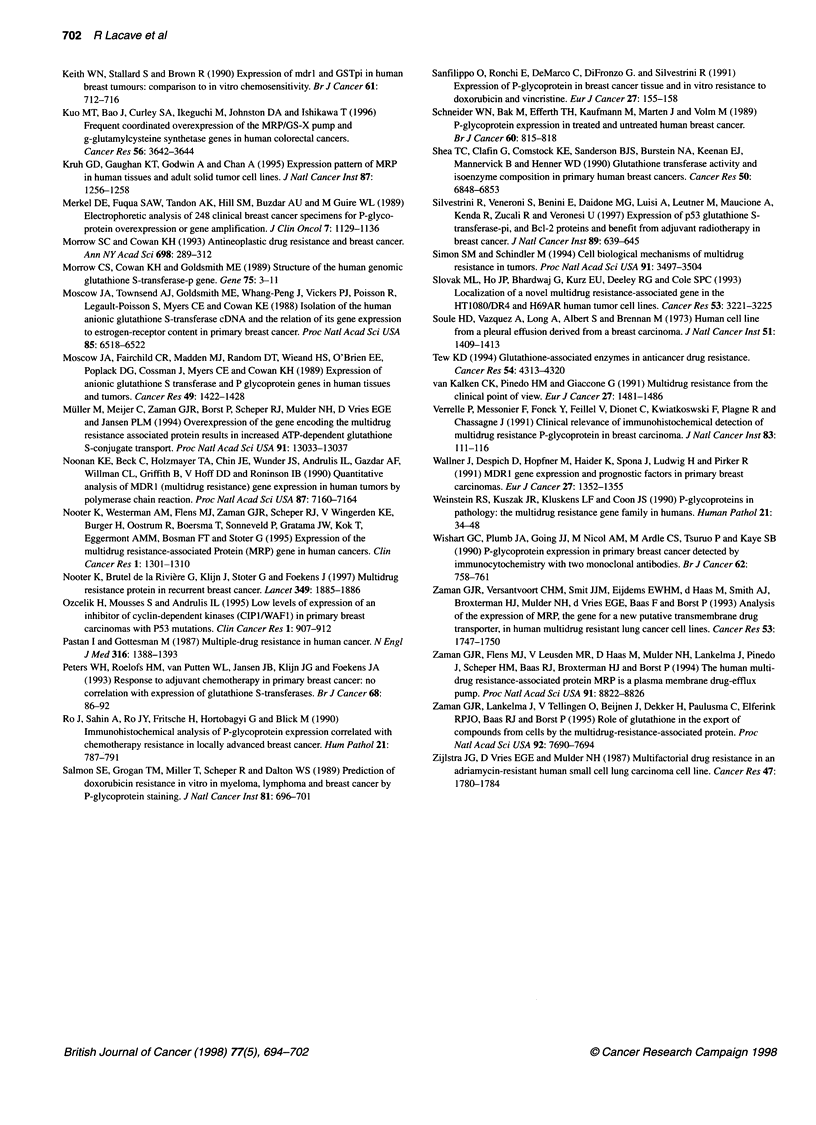


## References

[OCR_01042] Abbaszadegan M. R., Futscher B. W., Klimecki W. T., List A., Dalton W. S. (1994). Analysis of multidrug resistance-associated protein (MRP) messenger RNA in normal and malignant hematopoietic cells.. Cancer Res.

[OCR_01047] Akiyama S., Fojo A., Hanover J. A., Pastan I., Gottesman M. M. (1985). Isolation and genetic characterization of human KB cell lines resistant to multiple drugs.. Somat Cell Mol Genet.

[OCR_01052] Almquist K. C., Loe D. W., Hipfner D. R., Mackie J. E., Cole S. P., Deeley R. G. (1995). Characterization of the M(r) 190,000 multidrug resistance protein (MRP) in drug-selected and transfected human tumor cell.. Cancer Res.

[OCR_01057] Alvarez M., Paull K., Monks A., Hose C., Lee J. S., Weinstein J., Grever M., Bates S., Fojo T. (1995). Generation of a drug resistance profile by quantitation of mdr-1/P-glycoprotein in the cell lines of the National Cancer Institute Anticancer Drug Screen.. J Clin Invest.

[OCR_01063] Arceci R. J. (1993). Clinical significance of P-glycoprotein in multidrug resistance malignancies.. Blood.

[OCR_01067] Batist G., Tulpule A., Sinha B. K., Katki A. G., Myers C. E., Cowan K. H. (1986). Overexpression of a novel anionic glutathione transferase in multidrug-resistant human breast cancer cells.. J Biol Chem.

[OCR_01072] Beck W. T., Grogan T. M., Willman C. L., Cordon-Cardo C., Parham D. M., Kuttesch J. F., Andreeff M., Bates S. E., Berard C. W., Boyett J. M. (1996). Methods to detect P-glycoprotein-associated multidrug resistance in patients' tumors: consensus recommendations.. Cancer Res.

[OCR_01089] Breuninger L. M., Paul S., Gaughan K., Miki T., Chan A., Aaronson S. A., Kruh G. D. (1995). Expression of multidrug resistance-associated protein in NIH/3T3 cells confers multidrug resistance associated with increased drug efflux and altered intracellular drug distribution.. Cancer Res.

[OCR_01095] Brotherick I., Shenton B. K., Egan M., Cunliffe W. E., Browell D. A., Lunt L. G., Young J. R., Higgs M. J. (1996). Examination of multidrug resistance in cell lines and primary breast tumours by flow cytometry.. Eur J Cancer.

[OCR_01084] Bénard J., Da Silva J., De Blois M. C., Boyer P., Duvillard P., Chiric E., Riou G. (1985). Characterization of a human ovarian adenocarcinoma line, IGROV1, in tissue culture and in nude mice.. Cancer Res.

[OCR_01119] Charpin C., Vielh P., Duffaud F., Devictor B., Andrac L., Lavaut M. N., Allasia C., Horschowski N., Piana L. (1994). Quantitative immunocytochemical assays of P-glycoprotein in breast carcinomas: correlation to messenger RNA expression and to immunohistochemical prognostic indicators.. J Natl Cancer Inst.

[OCR_01125] Chevillard S., Pouillart P., Beldjord C., Asselain B., Beuzeboc P., Magdelénat H., Vielh P. (1996). Sequential assessment of multidrug resistance phenotype and measurement of S-phase fraction as predictive markers of breast cancer response to neoadjuvant chemotherapy.. Cancer.

[OCR_01101] Cole S. P., Bhardwaj G., Gerlach J. H., Mackie J. E., Grant C. E., Almquist K. C., Stewart A. J., Kurz E. U., Duncan A. M., Deeley R. G. (1992). Overexpression of a transporter gene in a multidrug-resistant human lung cancer cell line.. Science.

[OCR_01107] Cole S. P., Sparks K. E., Fraser K., Loe D. W., Grant C. E., Wilson G. M., Deeley R. G. (1994). Pharmacological characterization of multidrug resistant MRP-transfected human tumor cells.. Cancer Res.

[OCR_01112] Coste A., Rateau J. G., Roudot-Thoraval F., Chapelin C., Gilain L., Poron F., Peynegre R., Bernaudin J. F., Escudier E. (1996). Increased epithelial cell proliferation in nasal polyps.. Arch Otolaryngol Head Neck Surg.

[OCR_01131] Endicott J. A., Ling V. (1989). The biochemistry of P-glycoprotein-mediated multidrug resistance.. Annu Rev Biochem.

[OCR_01135] Flens M. J., Izquierdo M. A., Scheffer G. L., Fritz J. M., Meijer C. J., Scheper R. J., Zaman G. J. (1994). Immunochemical detection of the multidrug resistance-associated protein MRP in human multidrug-resistant tumor cells by monoclonal antibodies.. Cancer Res.

[OCR_01141] Flens M. J., Zaman G. J., van der Valk P., Izquierdo M. A., Schroeijers A. B., Scheffer G. L., van der Groep P., de Haas M., Meijer C. J., Scheper R. J. (1996). Tissue distribution of the multidrug resistance protein.. Am J Pathol.

[OCR_01146] Fojo A. T., Ueda K., Slamon D. J., Poplack D. G., Gottesman M. M., Pastan I. (1987). Expression of a multidrug-resistance gene in human tumors and tissues.. Proc Natl Acad Sci U S A.

[OCR_01151] Gilbert L., Elwood L. J., Merino M., Masood S., Barnes R., Steinberg S. M., Lazarous D. F., Pierce L., d'Angelo T., Moscow J. A. (1993). A pilot study of pi-class glutathione S-transferase expression in breast cancer: correlation with estrogen receptor expression and prognosis in node-negative breast cancer.. J Clin Oncol.

[OCR_01159] Gottesman M. M., Pastan I. (1993). Biochemistry of multidrug resistance mediated by the multidrug transporter.. Annu Rev Biochem.

[OCR_01168] Gregorcyk S., Kang Y., Brandt D., Kolm P., Singer G., Perry R. R. (1996). p-Glycoprotein expression as a predictor of breast cancer recurrence.. Ann Surg Oncol.

[OCR_01173] Hennequin E., Delvincourt C., Pourny C., Jardillier J. C. (1993). Expression of mdr1 gene in human breast primary tumors and metastases.. Breast Cancer Res Treat.

[OCR_01178] Horikoshi T., Danenberg K. D., Stadlbauer T. H., Volkenandt M., Shea L. C., Aigner K., Gustavsson B., Leichman L., Frösing R., Ray M. (1992). Quantitation of thymidylate synthase, dihydrofolate reductase, and DT-diaphorase gene expression in human tumors using the polymerase chain reaction.. Cancer Res.

[OCR_01185] Ishikawa T., Akimaru K., Kuo M. T., Priebe W., Suzuki M. (1995). How does the MRP/GS-X pump export doxorubicin?. J Natl Cancer Inst.

[OCR_01189] Jedlitschky G., Leier I., Buchholz U., Center M., Keppler D. (1994). ATP-dependent transport of glutathione S-conjugates by the multidrug resistance-associated protein.. Cancer Res.

[OCR_01200] Keith W. N., Stallard S., Brown R. (1990). Expression of mdr1 and gst-pi in human breast tumours: comparison to in vitro chemosensitivity.. Br J Cancer.

[OCR_01211] Kruh G. D., Gaughan K. T., Godwin A., Chan A. (1995). Expression pattern of MRP in human tissues and adult solid tumor cell lines.. J Natl Cancer Inst.

[OCR_01205] Kuo M. T., Bao J. J., Curley S. A., Ikeguchi M., Johnston D. A., Ishikawa T. (1996). Frequent coordinated overexpression of the MRP/GS-X pump and gamma-glutamylcysteine synthetase genes in human colorectal cancers.. Cancer Res.

[OCR_01216] Merkel D. E., Fuqua S. A., Tandon A. K., Hill S. M., Buzdar A. U., McGuire W. L. (1989). Electrophoretic analysis of 248 clinical breast cancer specimens for P-glycoprotein overexpression or gene amplification.. J Clin Oncol.

[OCR_01221] Morrow C. S., Cowan K. H. (1993). Antineoplastic drug resistance and breast cancer.. Ann N Y Acad Sci.

[OCR_01225] Morrow C. S., Cowan K. H., Goldsmith M. E. (1989). Structure of the human genomic glutathione S-transferase-pi gene.. Gene.

[OCR_01237] Moscow J. A., Fairchild C. R., Madden M. J., Ransom D. T., Wieand H. S., O'Brien E. E., Poplack D. G., Cossman J., Myers C. E., Cowan K. H. (1989). Expression of anionic glutathione-S-transferase and P-glycoprotein genes in human tissues and tumors.. Cancer Res.

[OCR_01229] Moscow J. A., Townsend A. J., Goldsmith M. E., Whang-Peng J., Vickers P. J., Poisson R., Legault-Poisson S., Myers C. E., Cowan K. H. (1988). Isolation of the human anionic glutathione S-transferase cDNA and the relation of its gene expression to estrogen-receptor content in primary breast cancer.. Proc Natl Acad Sci U S A.

[OCR_01244] Müller M., Meijer C., Zaman G. J., Borst P., Scheper R. J., Mulder N. H., de Vries E. G., Jansen P. L. (1994). Overexpression of the gene encoding the multidrug resistance-associated protein results in increased ATP-dependent glutathione S-conjugate transport.. Proc Natl Acad Sci U S A.

[OCR_01250] Noonan K. E., Beck C., Holzmayer T. A., Chin J. E., Wunder J. S., Andrulis I. L., Gazdar A. F., Willman C. L., Griffith B., Von Hoff D. D. (1990). Quantitative analysis of MDR1 (multidrug resistance) gene expression in human tumors by polymerase chain reaction.. Proc Natl Acad Sci U S A.

[OCR_01259] Nooter K., Westerman A. M., Flens M. J., Zaman G. J., Scheper R. J., van Wingerden K. E., Burger H., Oostrum R., Boersma T., Sonneveld P. (1995). Expression of the multidrug resistance-associated protein (MRP) gene in human cancers.. Clin Cancer Res.

[OCR_01266] Nooter K., de la Riviere G. B., Klijn J., Stoter G., Foekens J. (1997). Multidrug resistance protein in recurrent breast cancer.. Lancet.

[OCR_01270] Ozçelik H., Mousses S., Andrulis I. L. (1995). Low levels of expression of an inhibitor of cyclin-dependent kinases (CIP1/WAF1) in primary breast carcinomas with p53 mutations.. Clin Cancer Res.

[OCR_01275] Pastan I., Gottesman M. (1987). Multiple-drug resistance in human cancer.. N Engl J Med.

[OCR_01279] Peters W. H., Roelofs H. M., van Putten W. L., Jansen J. B., Klijn J. G., Foekens J. A. (1993). Response to adjuvant chemotherapy in primary breast cancer: no correlation with expression of glutathione S-transferases.. Br J Cancer.

[OCR_01286] Ro J., Sahin A., Ro J. Y., Fritsche H., Hortobagyi G., Blick M. (1990). Immunohistochemical analysis of P-glycoprotein expression correlated with chemotherapy resistance in locally advanced breast cancer.. Hum Pathol.

[OCR_01292] Salmon S. E., Grogan T. M., Miller T., Scheper R., Dalton W. S. (1989). Prediction of doxorubicin resistance in vitro in myeloma, lymphoma, and breast cancer by P-glycoprotein staining.. J Natl Cancer Inst.

[OCR_01297] Sanfilippo O., Ronchi E., De Marco C., Di Fronzo G., Silvestrini R. (1991). Expression of P-glycoprotein in breast cancer tissue and in vitro resistance to doxorubicin and vincristine.. Eur J Cancer.

[OCR_01302] Schneider J., Bak M., Efferth T., Kaufmann M., Mattern J., Volm M. (1989). P-glycoprotein expression in treated and untreated human breast cancer.. Br J Cancer.

[OCR_01307] Shea T. C., Claflin G., Comstock K. E., Sanderson B. J., Burstein N. A., Keenan E. J., Mannervik B., Henner W. D. (1990). Glutathione transferase activity and isoenzyme composition in primary human breast cancers.. Cancer Res.

[OCR_01313] Silvestrini R., Veneroni S., Benini E., Daidone M. G., Luisi A., Leutner M., Maucione A., Kenda R., Zucali R., Veronesi U. (1997). Expression of p53, glutathione S-transferase-pi, and Bcl-2 proteins and benefit from adjuvant radiotherapy in breast cancer.. J Natl Cancer Inst.

[OCR_01319] Simon S. M., Schindler M. (1994). Cell biological mechanisms of multidrug resistance in tumors.. Proc Natl Acad Sci U S A.

[OCR_01323] Slovak M. L., Ho J. P., Bhardwaj G., Kurz E. U., Deeley R. G., Cole S. P. (1993). Localization of a novel multidrug resistance-associated gene in the HT1080/DR4 and H69AR human tumor cell lines.. Cancer Res.

[OCR_01328] Soule H. D., Vazguez J., Long A., Albert S., Brennan M. (1973). A human cell line from a pleural effusion derived from a breast carcinoma.. J Natl Cancer Inst.

[OCR_01333] Tew K. D. (1994). Glutathione-associated enzymes in anticancer drug resistance.. Cancer Res.

[OCR_01341] Verrelle P., Meissonnier F., Fonck Y., Feillel V., Dionet C., Kwiatkowski F., Plagne R., Chassagne J. (1991). Clinical relevance of immunohistochemical detection of multidrug resistance P-glycoprotein in breast carcinoma.. J Natl Cancer Inst.

[OCR_01348] Wallner J., Depisch D., Hopfner M., Haider K., Spona J., Ludwig H., Pirker R. (1991). MDR1 gene expression and prognostic factors in primary breast carcinomas.. Eur J Cancer.

[OCR_01353] Weinstein R. S., Kuszak J. R., Kluskens L. F., Coon J. S. (1990). P-glycoproteins in pathology: the multidrug resistance gene family in humans.. Hum Pathol.

[OCR_01360] Wishart G. C., Plumb J. A., Going J. J., McNicol A. M., McArdle C. S., Tsuruo T., Kaye S. B. (1990). P-glycoprotein expression in primary breast cancer detected by immunocytochemistry with two monoclonal antibodies.. Br J Cancer.

[OCR_01375] Zaman G. J., Flens M. J., van Leusden M. R., de Haas M., Mülder H. S., Lankelma J., Pinedo H. M., Scheper R. J., Baas F., Broxterman H. J. (1994). The human multidrug resistance-associated protein MRP is a plasma membrane drug-efflux pump.. Proc Natl Acad Sci U S A.

[OCR_01379] Zaman G. J., Lankelma J., van Tellingen O., Beijnen J., Dekker H., Paulusma C., Oude Elferink R. P., Baas F., Borst P. (1995). Role of glutathione in the export of compounds from cells by the multidrug-resistance-associated protein.. Proc Natl Acad Sci U S A.

[OCR_01365] Zaman G. J., Versantvoort C. H., Smit J. J., Eijdems E. W., de Haas M., Smith A. J., Broxterman H. J., Mulder N. H., de Vries E. G., Baas F. (1993). Analysis of the expression of MRP, the gene for a new putative transmembrane drug transporter, in human multidrug resistant lung cancer cell lines.. Cancer Res.

[OCR_01386] Zijlstra J. G., de Vries E. G., Mulder N. H. (1987). Multifactorial drug resistance in an adriamycin-resistant human small cell lung carcinoma cell line.. Cancer Res.

[OCR_01337] van Kalken C. K., Pinedo H. M., Giaccone G. (1991). Multidrug resistance from the clinical point of view.. Eur J Cancer.

